# Comments on unresponsive decompression illness case

**DOI:** 10.1186/s40560-018-0347-z

**Published:** 2018-11-22

**Authors:** Bengusu Mirasoglu, Samil Aktas

**Affiliations:** Underwater and Hyperbaric Medicine Department, Istanbul Faculty of Medicine, Istanbul, Turkey

## Abstract

We have read the case report about a decompression sickness that was unresponsive to hyperbaric oxygen treatment in your journal. Presented case is intriguing; however, we think there are some contradictive issues in the discussion of the case. In this letter, we aim to comment on these issues that may raise further question. Bubble formation plays a very important role for decompression sickness, but proposed mechanism is incorrect as nitrogen does not change state during decompression. Use of terminology for diving-related diseases and comments on properties of helium may cause misunderstandings. Also importance of history of the dive in evaluating an accident should be emphasized.

## Letter

We read the case report entitled “A case of decompression illness not responding to hyperbaric oxygen” with great interest [1]. We totally agree with authors about multidisciplinary approach to decompression sickness. In our opinion, presence of a diving physician, an expert of diving diseases, in this team is crucial. Otherwise, unfortunate mistakes can be encountered. We would like to comment on some parts that we think may be misleading and incorrect from a diving medicine perspective.

### Definitions

We believe there is some confusion about the use of terminology all through the report. Authors have tried to distinguish decompression sickness (DCS), decompression illness (DCI), and arterial gas embolism (AGE) in the discussion section, but these explanations also have major flaws. In the report, authors divide DCI in two types as DCS and AGE and then claim that AGE results from bubbles entering arterial circulation via PFO. Shortly, DCS can be defined as the condition/disease caused by bubbles formed from dissolved gas in blood and/or tissue following an inadequate decompression [2]. Bubbles generally form in the tissues or venous system; however, in case of excessive supersaturation, arterial bubble formation is also possible. Arterialization of venous bubbles is possible in the presence of PFO (or right to left shunt); however, this does not change the name of the disease [3]. Either by bubble formation in the arterial system or arterialization from a right to left shunt, this condition is not named AGE, it is DCS. On the other hand, AGE is considered a form of barotrauma in diving medicine. Barotrauma is totally a different entity and defines the injury that results from volume changes of gases during ambient pressure change according to Boyle Gas Law. AGE is the term used for embolization of air from lungs as a result of overexpansion and rupture of pulmonary tissue by the air that is not exhaled during rapid ascent. That said, DCI is the term used for all diseases and conditions that can occur during decompression [2, 4].

### Pathophysiology

One very important point we think that should be mentioned is the mechanism that authors propose for explaining bubble formation. We think it is incorrect. Nitrogen does not change from liquid phase to gas phase during ascent. Change of state of matter (or phase change) is a very different subject and do not occur during diving. Mechanism of gas exchange during diving is explained by Henry Gas Law and is related to change of dissolving of gas with pressure, not the state of matter. When there is inadequate decompression, dissolved gas cannot be eliminated and forms bubbles.

We also think that the paragraph about the use of helium in the conclusion section may have some inconsistencies. Authors propose replacing nitrogen with helium for decreasing narcotic effects and bubble formation but then indicate that helium can be narcotic at high concentrations. Generally, helium is known to be non-narcotic, but only in a few articles mood and behavior changes have been reported at high pressures, not high concentrations. Thus, helium narcosis is more of a theoretical entity and attributing a narcotic effect to helium in relation to concentration may be misleading [5]. Furthermore, “less bubble formation with helium” is arguable. In fact, need for decompression stops may be more with shorter bottom times at certain depths. Also, relating bubble formation only to gas density would be inadequate as many other factors such as solubility, diffusion coefficient, tissue circulation, or surface tension of the bubble are important [6, 7].

### Medical management

Dive history is essential for the diagnosis of diving-related diseases, so diving profiles should be obtained in detail. In this case report, diving history is given very briefly: three consecutive dives to 40 m with 30 min time each. However, data like bottom time of the dives, surface intervals, decompression stops if performed by the diver, ascent time of the last dive, or breath holding during rapid ascent in this dive would have been guiding for diagnosis if they had been presented by the authors. Without these details about the dives, it may be hard to speculate on the clinical situation.

In addition, we would like to make one comment on medical management of this case. As authors have proposed, this patient may be suffering from AGE. In such a case, it would be suggested to evaluate the patient in terms of pulmonary barotrauma and pathologies that may lead to it such as air trapping lesions in the lungs. A thorax CT imaging would be a helpful and easily accessible tool. We read that the patient was evaluated by a CT angiography which may provide thoracic investigation too. So it is possible the authors have evaluated the patient in that regard but they have not mentioned in the report.

## Abbreviations

AGE: Arterial gas embolism
CT: Computed tomography
DCI: Decompression illness
DCS: Decompression sickness
